# Cardiovascular effects of 6-nitrodopamine, adrenaline, noradrenaline, and dopamine in normotensive and hypertensive rats

**DOI:** 10.3389/fphar.2025.1557997

**Published:** 2025-05-20

**Authors:** Vivian Fuguhara, Mariana Gonçalves De Oliveira, Carlos Alberto Aguiar da Silva, Pedro Renato Guazzelli, Larryn W. Peterson, Gilberto De Nucci

**Affiliations:** ^1^ Department of Pharmacology, Faculty of Medical Science, State University of Campinas (UNICAMP), Campinas, Brazil; ^2^ Laboratory of Multidisciplinary Research, São Franscisco University (USF), Bragança Paulista, Brazil; ^3^ Department of Physiology, Ribeirão Preto Medical School, University of São Paulo, Ribeirão Preto, Brazil; ^4^ Department of Chemistry, Rhodes College, Memphis, TN, United States; ^5^ Department of Pharmacology, Institute of Biomedical Sciences, University of São Paulo (ICB-USP), São Paulo, Brazil; ^6^ Deparment of Pharmacology, Faculty of Medicine, Campinas, Brazil; ^7^ Department of Pharmacology, Faculty of Medicine, Metropolitan University of Santos, Santos, Brazil

**Keywords:** L-NAME, nitrocatecholamines, monoamine oxidase, enzyme immunoassay, nitric oxide

## Abstract

**Introduction:**

6-Nitrodopamine (6-ND) has been extensively investigated using in vitro protocols, especially in the cardiovascular system. Despite the established more potent positive chronotropic and inotropic effects, in comparison with the classical catecholamines, adrenaline (ADR), noradrenaline (NA) and dopamine (DA), the effects with in vivo models are not determined yet. Here, we investigated the acute effects on heart rate (HR) and mean arterial blood pressure (MABP) in normotensive and hypertensive rats by 6-ND.

**Methods:**

Adult male Wistar rats were randomly divided into two groups: one received regular filtered water and the other a chronic Nω-nitro-L-arginine methyl ester (L-NAME) treatment in the water (20mg/day/rat, 4weeks). Thereafter, the animals were anesthetized, the HR and MABP were monitored through the femoral artery, and the vein was used to administer bolus injections of 6-ND, ADR, NA and DA. Moreover, 6-ND, classical and novel catecholamines, 6-cyanodopamine (6-CYANO), 6nitroadrenaline (6-NADR), and 6-nitrodopa (6-NDOPA) were used to assess their effect on monoamine oxidase (MAO) activity.

**Results:**

All four drugs significantly increased the HR in control animals; 6-ND was more potent than the classical catecholamines and its positive chronotropic effect was abolished with the chronic L-NAME treatment. Furthermore, in a cell-free assay, 6-ND was able to partially inhibit MAO-A (<30% at 1 mM) and MAO-B (<40% at 10 μM); 6-NDOPA and 6-CYANO (1 mM) inhibited MAO-A, and MAO-B by around 40%, respectively. ADR produced MAO-A inhibition of <20%, at 100μM.

**Conclusions:**

These results clearly demonstrate that 6-ND is the most potent endogenous positive chronotropic agent yet described, and its effects are independent of MAO inhibition.

## 1 Introduction

Nitrocatecholamines were found endogenously in rats, such as nitronoradrenaline and nitroadrenaline observed in brain extracts (Tsunoda et al., 2007), and 6-nitronoradrenaline released *in vivo* and detected in microdialysates of the dorsal horn of the spinal cord (Chiari et al., 2000). Additionally, rat isolated right atrium (Britto-Júnior et al., 2022a) and rat isolated ventricles (Britto-Júnior et al., 2023a) present basal release of 6-nitrodopamine (6-ND). In the isolated right atrium, 6-ND is a potent positive chronotropic agent, being 100 times more potent than adrenaline (ADR) and noradrenaline (NA), and 10,000 times more potent than dopamine (DA) (Britto-Júnior et al., 2022a). In the Langendorff preparation of the rat isolated heart, 6-ND also presents positive inotropic action, being more potent than classical catecholamines (Britto-Júnior et al., 2023a). Isolated vascular tissues, such as human umbilical cord vessels (Britto-Júnior et al., 2021a), human popliteal artery and vein (de Oliveira et al., 2023), also present basal release of 6-ND. In vascular tissues, 6-ND acts as a potent vasorelaxant, and its mechanism of action is associated with antagonism of dopamine D2-receptors (Britto-Júnior et al., 2021a).

The synthesis/release of 6-ND is coupled to NO synthesis. Isolated vascular tissues (Britto-Júnior et al., 2022a; Campos et al., 2021; Britto-Júnior et al., 2023b), rat isolated heart (Britto-Júnior et al., 2023a), and rat (Britto-Júnior et al., 2021b) and human vas deferens (Britto-Júnior et al., 2022b) released significantly lower amounts of 6-ND when they were pre-treated (30 min) with L-NAME. Nitric oxide can “nitrate” catecholamines *in vitro* (d’Ischia and Costantini, 1995); however, how this process occurs *in vivo* is not clear. It apparently involves the endothelial peroxidase NOX-1,4, since pre-treatment of tissues with the NOX inhibitors GKT-137831 (Aoyama et al., 2012) and diphenyleneiodonium (DPI) (Szilagyi et al., 2016; Li and Rand, 1996), increases the basal release of 6-ND (Britto-Júnior et al., 2024).

Although the *in vitro* action of 6-ND in the cardiovascular system has been extensively investigated (Zatz and De Nucci, 2024), to date, studies with 6-ND *in vivo* are scarce (Britto-Júnior et al., 2022a). Here, it is reported the effects of intravenous injections of 6-ND on the heart rate and blood pressure of anaesthetized control rats and in rats that were chronically treated with L-NAME (Ribeiro et al., 1992). The cardiovascular effects of intravenous administration of 6-ND were compared with those produced by injections of noradrenaline, adrenaline, and dopamine.

## 2 Materials and methods

### 2.1 Ethical aspects

All experimental procedures followed the ARRIVE guidelines 2.0 (du Sert et al., 2020), the Brazilian Guidelines for the Use of Animals from the National Council of Control in Animal Experimentation (CONCEA) (Conselho Nacional de Controle de Experimentação Animal, 2023) and were in accordance with the Guide for the Care and Use of Laboratory Animals from the National Institutes of Health (NIH Guidelines) (National Research Council, 2011). The protocols were approved by the Ethics Committee for Animal Use of State University of Campinas (CEUA/UNICAMP; Protocol No. 5942-1/2022, 6205-1/2023, 6229-1/2023, 6320-1/2023).

### 2.2 Animals

Adult male Wistar rats were provided by the Multidisciplinary Center for Biological Investigation on Laboratory Animal Science - CEMIB, University of Campinas (CEMIB/UNICAMP; Brazil) and maintained in the Pharmacology Bioterium (FCM/UNICAMP). Three animals were housed per cage, with a 12 h light-dark cycle, the humidity was maintained at 55% ± 5% and the temperature in 24°C ± 1°C. During the whole study, the animals received water and standard rodent food *ad libitum*.

### 2.3 Chronic L-NAME treatment

After 14 days of acclimatization, a group of animals was randomly selected and treated for at least 4 weeks with Nω-nitro-L-arginine methyl ester (L-NAME), a nitric oxide (NO) synthase inhibitor, dissolved in the filtered drinking water (20 mg/rat/day) (Ribeiro et al., 1992). To monitor the development of hypertension, the blood pressure (BP) and heart rate (HR) were monitored at two time points before (day 1 for control) and 4 weeks (day 28 for control) after the administration of L-NAME, using the non-invasive blood pressure analyzer (BP-2000 Blood Pressure Analysis System™, Visitech Systems), for the control group. This monitoring was performed randomly in some animals.

### 2.4 Invasive measurement of the heart rate (HR) and mean arterial blood pressure (MABP) in anaesthetized rats

Control (n = 109) and chronic L-NAME treated (n = 56) animals (10–16 weeks old) were sedated with isoflurane 5% for 1 min, followed by anesthesia with sodium thiopental (40 mg/kg, i.p.) and ketamine (70 mg/kg, i.p.). A skin incision was performed on the right leg to expose the right femoral vein and artery. After isolating, these vessels were canulated with a polyethylene PE10 catheter filled with heparinized saline solution (50 IU/mL heparin in 0.9% saline). Heparin was administered (600 UI/kg, s.c.) to avoid blood clots or register interruptions. The HR and MABP were monitored through the artery catheter, which was coupled to a pressure transducer (MLT0699 Disposable BP Transducer, AD Instruments, Sidney, Australia). Data was acquired by a computerized system (PowerLab, AD Instruments) using acquisition software (LabChart, AD Instruments). The HR was derived from the pulse pressure and expressed as beats per minute (bpm). The MABP (expressed in mmHg) was calculated by the software considering 1/3 Maximum + 2/3 Minimum (1/3 systolic + 2/3 diastolic). During the whole experiment anesthetic depth was assessed by muscle relaxation, the loss of response of stimulation to toe pinch, observation of the movement of the chest and the HR and MABP. Additional doses of anesthetics were administered as necessary.

### 2.5 Drug administration

All the drugs were diluted in 0.9% saline solution and administered through the femoral vein catheter. After stabilization for at least 20 min, animals received either 6-ND (0.1, 0.3, 1 and 10 pmol/kg), ADR (10, 30 and 100 pmol/kg), NA (0.3, 1 and 3 nmol/kg), DA (1, 10, 100 and 300 nmol/kg), or the vehicle (0.9% saline; 25 μL) through intravenous bolus. The volume of the injected drugs varied from 15 to 23 μL, which considers the catheter length; after the drug injection, the catheter was flushed with 25 μL of saline. The HR and MABP were monitored for 30 min following drug injection. One animal was administered only one dose of one catecholamine. At the end of the experiments, the rats were euthanized with an overdose of anesthetic (sodium thiopental >100 mg/kg, i.v.). Exsanguination was performed to confirm euthanasia.

### 2.6 Monoamine oxidase activity

MAO-A and B inhibition were assessed using a MAO Inhibitor Screening Kit (MAK295 and MAK296, for MAO-A and MAO-B, respectively, both from Sigma Aldrich, St. Louis, United States) following the manufacturer’s instructions. This cell-free assay relies on the fluorometric detection of hydrogen peroxide (H_2_O_2_), a by-product generated during the oxidative deamination of the MAO substrate (tyramine). Various concentrations (ranging from 100 nM to 1 mM) of 6-nitrodopamine (6-ND), adrenaline (ADR), noradrenaline (NA), dopamine (DA), 6-cyanodopamine (6-CYANO), 6-nitroadrenaline (6-NADR), and 6-nitrodopa (6-NDOPA) were tested in duplicate. Clorgyline and selegiline were employed as positive controls for MAO-A and MAO-B, respectively, at the same concentrations of the tested catecholamines. Fluorescence (excitation = 535 nm/emission = 587 nm) was determined by kinetic reading between 10 and 30 min. Autofluorescence testing was carried out in duplicate for each catecholamine, and the results were negative (data not shown). Two points (T1 and T2) in the linear range of the plot were chosen, and the corresponding fluorescence values (RFU1 and RFU2) were obtained. The results were plotted as drug concentration relative to the percentage (%) of relative MAO activity.

### 2.7 Drugs

6-Nitrodopamine (6-ND) and 6-nitroadrenaline (6-NADR) were purchased from Toronto Research Chemicals Inc. (Toronto, Ontario, Canada). Adrenaline (ADR), noradrenaline (NA), dopamine (DA), and Nω-nitro-L-arginine methyl ester (L-NAME) were acquired from Cayman Chemical Co. (Michigan, United States). Sodium thiopental (Thiopentax) and sodium heparin (HEMefol) were purchased from Cristália (Itapira, Brazil). Ketamine was acquired from Dechra (Londrina, Brazil). 6-Cyanodopamine (6-CYANO; 97.8% purity) and 6-nitrodopa (6-NDOPA; 93.8% purity) were synthesized in house (Rote et al., 2017; Nyamkondiwa et al., 2022; Ribeiro et al., 2024). Clorgyline and selegiline were provided in the MAO assay kits.

### 2.8 Statistical analysis

Data are expressed as mean ± standard error of the mean (SEM). Paired Student’s t-test was used to compare the basal values each 5 minutes after bolus administration at the same dose of a drug. A significance level of 5% was considered (p < 0.05). For data analysis and graphics generation were used Microsoft Excel 2016 and GraphPad Prism 8.4.3.

## 3 Results

### 3.1 Basal HR, MABP, systolic blood pressure, diastolic blood pressure, and pulse pressure in conscious control animals

The HR and MABP of control animals were 374 ± 8 bpm and 101 ± 4 mmHg, respectively, in day 1, and 364 ± 5 bpm (p = 0.19 vs. day 1) and 92 ± 3 mmHg (p = 0.060 vs. day 1) after 28 days. The systolic pressure on day 1 and day 28 were 144 ± 4 mmHg and 135 ± 3 mmHg (p = 0.07), respectively. The diastolic blood pressure on day 1 and day 28 were 79 ± 5 mmHg and 70 ± 3 mmHg (p = 0.09), respectively, and the pulse pressure were 65 ± 3 and 66 ± 3 (p = 0.89). Data generated by non-invasive monitoring in control animals are described in the Supplementary Figures S7A–E.

### 3.2 Basal HR, MABP, systolic blood pressure, diastolic blood pressure, and pulse pressure in conscious L-NAME chronically treated animals

L-NAME chronically treated animals were 389 ± 5 bpm and 93 ± 2 mmHg, before L-NAME treatment, and 336 ± 3 bpm (p < 0.001 vs. before treatment) and 163 ± 3 mmHg (p < 0.001 vs. before treatment) after L-NAME treatment. The systolic pressure before and after L-NAME treatment were 139 ± 3 mmHg and 197 ± 3 mmHg (p < 0.001), respectively. The diastolic blood pressure before and after L-NAME treatment were 69 ± 2 and 147 ± 4 mmHg (p < 0.001), respectively, and the pulse pressure were 69 ± 3 and 50 ± 3 mmHg (p < 0.001), respectively. Data generated by non-invasive monitoring in L-NAME chronically treated animals are described in the Supplementary Figures S8A–E.

### 3.3 Basal HR, MABP, systolic blood pressure, diastolic blood pressure, and pulse pressure in anesthetized animals

The HR and MABP of anesthetized animals were 366 ± 4 bpm and 105 ± 1 mmHg, respectively, from control rats, and 391 ± 6 bpm (p < 0.001 vs. control) and 161 ± 2 mmHg (p < 0.001 vs. control), respectively, from L-NAME chronically treated rats. The systolic blood pressure of anesthetized animals was 131 ± 1 mmHg and 192 ± 2 (p < 0.001) in control and L-NAME chronically treated animals. The diastolic blood pressure of anesthetized animals was 91 ± 1 and 146 ± 2 mmHg (p < 0.001) in control and L-NAME chronically treated animals, respectively. The pulse pressures were 40 ± 1 and 46 ± 1 mmHg (p < 0.001) in control and L-NAME chronically treated animals, respectively.

### 3.4 Effect of 6-ND, ADR, NA, and DA injections on the HR of control rats

**FIGURE 1:**
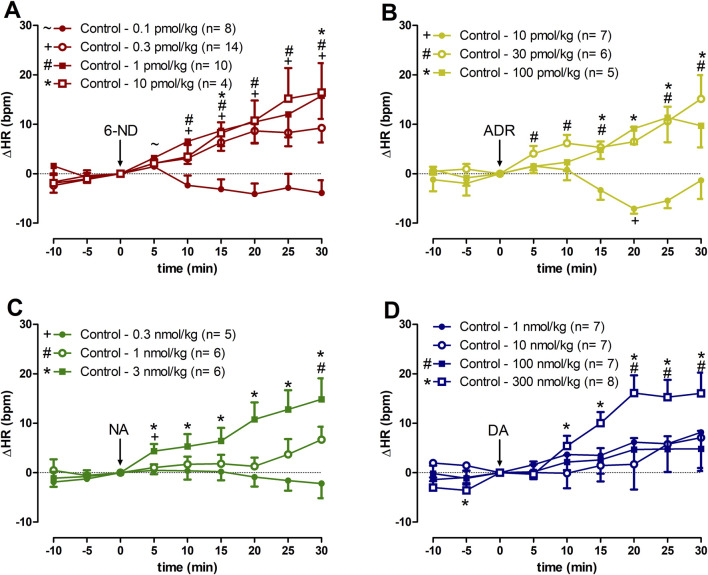
Increases of heart rate (∆HR) induced by 6-nitrodopamine and classical catecholamines in anaesthetized control rats. Intravenous bolus injections of 6-nitrodopamine (**(A)**, 6-ND; 0.1–10 pmol/kg), adrenaline (**(B)**, ADR; 10–100 pmol/kg), noradrenaline (**(C)**, NA; 0.3–3 nmol/kg), and dopamine (**(D)**, DA; 1–300 nmol/kg) were monitored for 30 min. The characters “∼, +, # and *” and their position, above or below the x-axis, indicate p < 0.05 in comparison with the point “0”, when the drug was injected. Paired Student’s t-test was used to compare the basal values each 5 minutes after bolus administration at the same dose of a drug.

**FIGURE 2:**
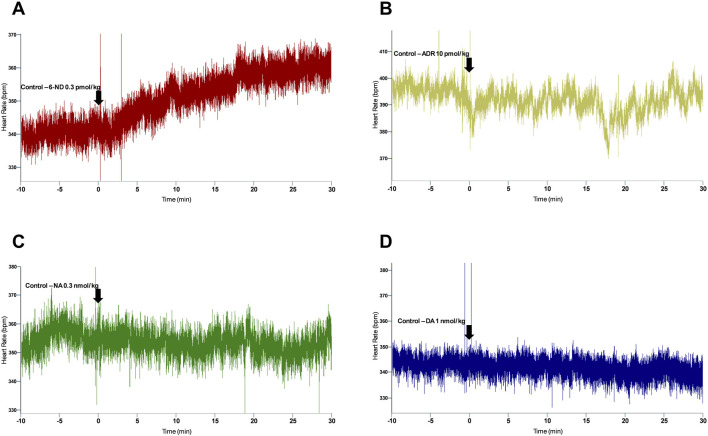
Representative tracing of the heart rate responses to bolus injection of 6-ND (**(A)**, 0.3 pmol/kg), ADR (**(B)**, 10 pmol/kg), NA (**(C)**, 0.3 nmol/kg), and DA (**(D)**, 1 nmol/kg) in control rats, showing a potency comparison of 6-ND with classical catecholamines.

Bolus injections of 6-ND (0.1 pmol/kg) did not affect the HR (Figure 1A). However, bolus injections of higher doses of 6-ND (0.3, 1, and 10 pmol/kg) caused significant increases in the HR. Bolus injections of ADR (10 pmol/kg) did not cause an increase in the HR (Figure 1B). Bolus injections of higher doses of ADR (30 and 100 pmol/kg) significantly increased the HR. Bolus injections of NA (0.3 and 1 nmol/kg) caused no alterations in HR (Figure 1C). Bolus injections of 3 nmol/kg NA caused significant increases in HR. Bolus injections of DA (1, 3, and 100 nmol/kg) did not increase HR (Figure 1D). Bolus injection of DA (300 nmol/kg) caused a significant increase in HR. A representative tracing illustrating the higher potency of 6-ND in comparison with the classical catecholamines ADR, NA and DA is shown in Figure 2.

### 3.5 Effect of 6-ND, ADR, NA, and DA injections on the HR of L-NAME chronically treated rats

**FIGURE 3:**
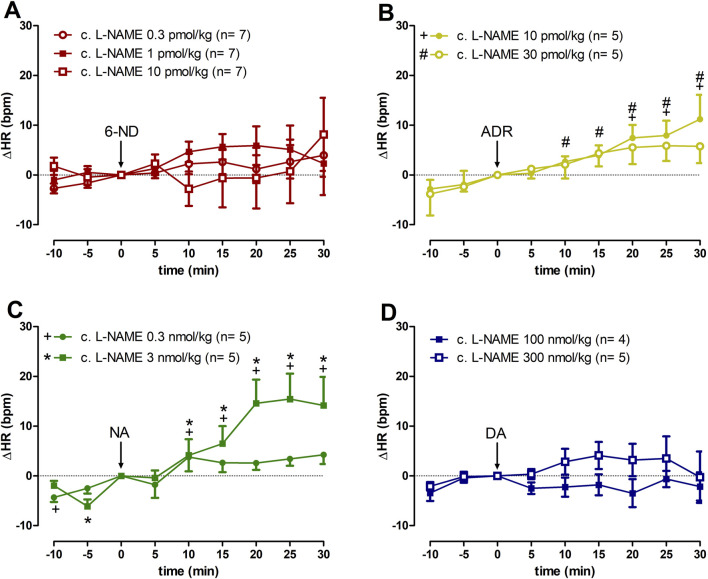
Increases of heart rate (∆HR) induced by 6-nitrodopamine and classical catecholamines in L-NAME chronically treated rats. Intravenous bolus injections of 6-nitrodopamine (**(A)**: 6-ND; 0.3–10 pmol/kg); adrenaline (**(B)**, ADR; 10 and 30 pmol/kg); noradrenaline (**(C)**, NA; 0.3 and 3 nmol/kg); and dopamine (**(D)**, DA; 100 and 300 nmol/kg) were monitored for 30 min. The characters “+, # and *” and their position, above or below the x-axis, indicate p < 0.05 in comparison with the point “0”, when the drug was injected. Paired Student’s t-test was used to compare the basal values each 5 minutes after bolus administration at the same dose of a drug.

In animals chronically treated with L-NAME, bolus injections of 6-ND (0.3, 1, and 10 pmol/kg) did not cause significant increases in the HR (Figure 3A). In contrast to 6-ND, bolus injections of ADR (10 and 30 pmol/kg) caused significant increases in the HR (Figure 3B). Bolus injections of NA (0.3 and 3 nmol/kg) induced significant increases in HR (Figure 3C). Bolus injections of DA (100 and 300 nmol/kg) did not induce significant changes in the HR (Figure 3D).

### 3.6 Effect of 6-ND, ADR, NA, and DA injections on the MABP of control rats

**FIGURE 4:**
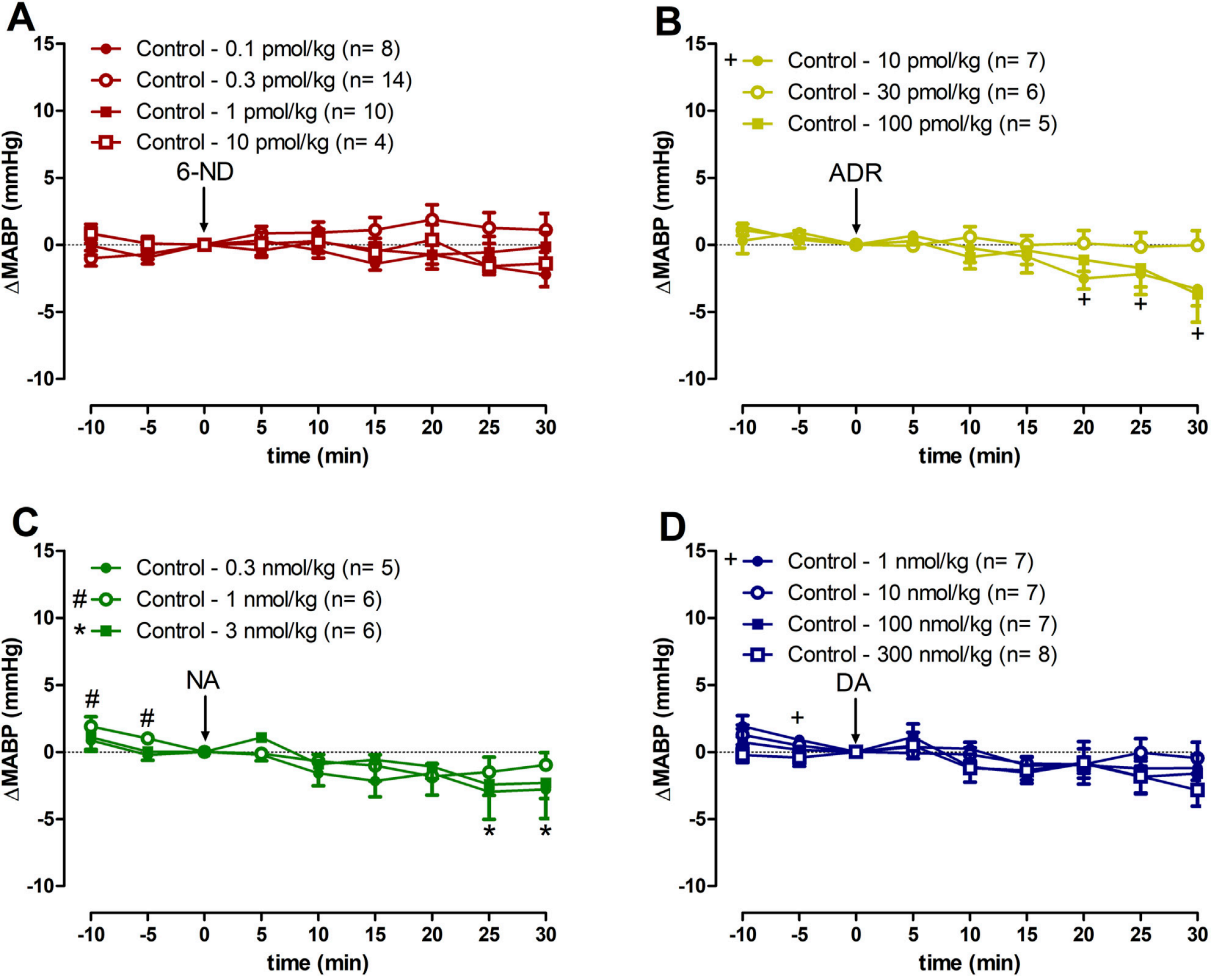
Changes of the mean arterial blood pressure (∆MABP) induced by 6-nitrodopamine and classical catecholamines in anaesthetized control rats. Intravenous bolus injections of 6-nitrodopamine (**(A)**, 6-ND; 0.1–10 pmol/kg), adrenaline (**(B)**, ADR; 10–100 pmol/kg), noradrenaline (**(C)**, NA; 0.3–3 nmol/kg), and dopamine (**(D)**, DA; 1–300 nmol/kg) were monitored for 30 min. The characters “+, # and *” and their position, above or below the x-axis, indicate p < 0.05 in comparison with the point “0”, when the drug was injected. Paired Student’s t-test was used to compare the basal values each 5 minutes after bolus administration at the same dose of a drug.

Bolus injections of 6-ND (0.1–10 pmol/kg) had no effect in the MABP (Figure 4A). Bolus injections of ADR 10 pmol/kg caused a minor but significant decrease in MABP 15 min after the injection (Figure 4B), which was not observed at higher doses (30 and 300 pmol/kg). Bolus injections of NA (0.3 and 1 nmol/kg) did not cause significant alterations in MABP, whereas bolus injections of higher dose (3 nmol/kg) caused minor but significant falls in MABP 25 min after the injection (Figure 4C). Bolus injections of DA (1–300 nmol/kg) did not induce significant changes in blood pressure (Figure 4D). Data generated by 6-ND administration on the systolic blood pressure, diastolic blood pressure, and pulse pressure in control animals are described in the Supplementary Figures S1A–C, respectively. Supplementary Figures S2–S4 contain the data on the systolic blood pressure, diastolic blood pressure, and pulse pressure in control animals generated by ADR, NA, and DA injections, respectively.

### 3.7 Effect of 6-ND, ADR, NA, and DA injections on the MABP of L-NAME chronically treated rats

**FIGURE 5:**
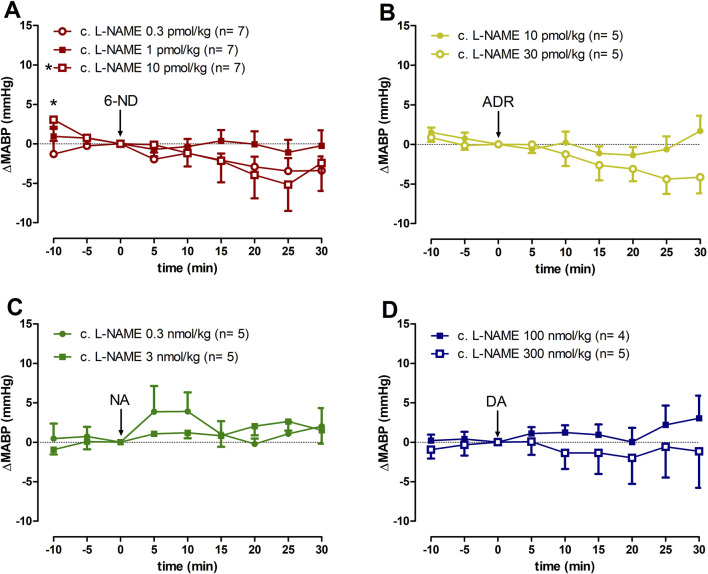
Changes of mean arterial blood pressure (∆MABP) induced by 6-nitrodopamine and classical catecholamines in L-NAME chronically treated rats. Intravenous bolus injections of 6-nitrodopamine (**(A)**, 6-ND; 0.3–10 pmol/kg), adrenaline (**(B)**, ADR; 10 and 30 pmol/kg), noradrenaline (**(C)**, NA; 0.3 and 3 nmol/kg), and dopamine (**(D)**, DA; 100 and 300 nmol/kg) in the anaesthetized chronically treated with L-NAME rat. The character “*” and its position, above the x-axis, indicate p < 0.05 in comparison with the point “0”, when the drug was injected. Paired Student’s t-test was used to compare the basal values each 5 minutes after bolus administration at the same dose of a drug.

Bolus injections of 6-ND (0.3, 1, and 10 pmol/kg; Figure 5A), ADR (10 and 30 pmol/kg; Figure 5B), NA (0.3 and 3 nmol/kg; Figure 5C), and DA (100 and 300 nmol/kg; Figure 5D) caused no significant changes in the MABP. Data generated by 6-ND administration on the systolic blood pressure, diastolic blood pressure, and pulse pressure in L-NAME chronically treated animals are described in the Supplementary Figures S1D–F, respectively. Supplementary Figures S2–S4 contain the data on the systolic blood pressure, diastolic blood pressure, and pulse pressure in L-NAME chronically treated animals generated by ADR, NA and DA injections, respectively.

### 3.8 Effect of saline injection on the HR and MABP of control and L-NAME chronically treated rats

Bolus injections of saline (25 μL) had no effect on HR (Supplementary Figure S5A) and MABP (Supplementary Figure S5B) of either control or L-NAME chronically treated rats. Data generated by saline administration on the systolic blood pressure, diastolic blood pressure, and pulse pressure in control and in L-NAME chronically treated animals are described in the Supplementary Figures S6A–C, respectively.

### 3.9 Effect of 6-ND, 6-NDOPA, 6-CYANO, 6-NADR, ADR, NA, and DA in MAO-A activity

The control MAO-A inhibitor, clorgyline, inhibited >80% at 1 μM, and almost 100% at higher concentrations. 6-ND and 6-NDOPA produced an inhibition of <30% and around 40%, respectively, with the highest concentration (1 mM), while ADR produced an inhibition <20% at 100 μM and 1 mM. NA, DA, 6-CYANO, and 6-NADR did not present significant effects on MAO-A activity (Table 1).

**TABLE 1:** Percent activity reduction of monoamine oxidase A (MAO-A) relative to no inhibitor (%) by clorgyline, 6-nitrodopamine (6-ND), adrenaline (ADR), noradrenaline (NA), dopamine (DA), 6-cyanodopamine (6-CYANO), 6-nitroadrenaline (6-NADR), and 6-nitrodopa (6-NDOPA).

MAO-A activity (%)					
	100 nM	1 µM	10 µM	100 µM	1 mM
Clorgyline	83.01	16.35	2.42	0.27	0.17
6-ND	100	90.60	97.42	88.65	78.03
ADR	100	97.15	94.94	81.90	81.94
NA	100	97.90	96.81	96.81	96.81
DA	100	97.55	92.34	91.49	84.36
6-CYANO	100	95.99	92.45	97.17	90.31
6-NADR	100	98.40	90.55	93.8	95.80
6-NDOPA	100	100	94.82	90.25	58.81

### 3.10 Effect of 6-ND, 6-NDOPA, 6-CYANO, 6-NADR, ADR, NA, and DA in MAO-B activity

The control MAO-B inhibitor, selegiline, presented an inhibition of approximately 70% at 1 μM, and almost 100% at higher concentrations. 6-ND and 6-CYANO produced <40% MAO-B inhibition at 1 mM, whereas ADR, NA, DA, 6-NADR, and 6-NDOPA did not present significant effects on MAO-B activity (Table 2).

**TABLE 2:** Percent activity reduction of monoamine oxidase B (MAO-B) relative to no inhibitor (%) by selegiline, 6-nitrodopamine (6-ND), adrenaline (ADR), noradrenaline (NA), dopamine (DA), 6-cyanodopamine (6-CYANO), 6-nitroadrenaline (6-NADR), and 6-nitrodopa (6-NDOPA).

MAO-B activity (%)					
	100 nM	1 µM	10 µM	100 µM	1 mM
Selegiline	96.96	27.74	1.39	2.34	1.34
6-ND	95.78	88.07	64.32	77.75	67.38
ADR	94.55	88.77	95.75	100	100
NA	83.9	87.55	89.86	100	100
DA	98.47	90.93	100	100	100
6-CYANO	96.32	87.01	79.47	74.85	60.47
6-NADR	100	100	100	97.69	93.41
6-NDOPA	96.79	95.14	87.03	91.73	89.64

## 4 Discussion

The results clearly demonstrate that 6-ND is the most potent endogenous positive chronotropic agent yet described, both *in vitro* (Britto-Júnior et al., 2022a; Britto-Júnior et al., 2023a) and now *in vivo*. Activation of β-adrenoreceptor leads to stimulation of adenylyl cyclase (Brodde, 1988), indicating a major role of cyclic AMP as a modulator of heart rate (Tomek and Zaccolo, 2023). Indeed, inhibition of cAMP-dependent protein kinase A (PKA) induces substantial bradycardia in cardiac pacemaker cells (Vinogradova et al., 2006), and independent of either PDE3 or PDE4, induces sinoatrial bradycardia in the rat and mouse (Galindo‐Tovar and Kaumann, 2008). Although 6-ND is a catecholamine and causes increases in heart rate, its mechanism of action is distinct from the classical catecholamine (Britto-Júnior et al., 2023c). The use of an agarose bead coupled to 6-nitrodopamine for purification of human cardiomyocyte membranes revealed selective binding to three proteins that modulate adenylyl cyclase, such as cyclase associated protein 1 (CAP1), cyclase associated protein 2 (CAP2), and stromal interaction protein 1 (STIM1) (Sweed et al., 2024). Cyclase associated protein 1 (CAP1) binds and activates adenylyl cyclase in mammalian cells (Zhang et al., 2021). Ablation of CAP2 in mice causes dilated cardiomyopathy associated with severe reduction of the heart rate (Peche et al., 2017). Stromal interaction protein (STIM1) is expressed in cardiomyocytes (Peche et al., 2017; Liu et al., 2023), has a single transmembrane domain (Hooper et al., 2000), is in the sarcoplasmic reticulum and plasma membrane (Soboloff et al., 2012), and it is also associated with adenylyl cyclase activation (Motiani et al., 2018). These proteins are the main candidates for the proposed 6-ND receptor mediated modulation of adenylyl cyclase. Thus, it is possible that the positive chronotropic effect induced by 6-ND could be associated with its action on cyclase-associated proteins.

Inhibition of monoamine oxidase in the rat isolated atria is associated with potentiation of the positive chronotropic effect induced by NA (Murnaghan, 1968). Apigenin blocks both MAO-A and MAO-B (Chaurasiya et al., 2014), and it causes concentration-dependent increases in rat isolated basal atrial rate (Lorenzo et al., 1996). At a high concentration (1 mM), 6-ND inhibits MAO-B (Huotari et al., 2001). As MAO-A preferentially metabolizes NA (Johnston, 1968), inhibition of MAO-A by 6-ND could be a possible positive chronotropic mechanism. Although in humans, DA is oxidized by MAO-B (Glover et al., 1977), and in rodents, DA is oxidized by MAO-A (Neff and Yang, 1974). However, the finding that 6-ND produced <30% of MAO-A inhibition at 1 mM indicates that is doubtful that this inhibition could contribute to its positive chronotropic effect.

Nitric oxide inhibition is associated with a reduction in basal release of 6-ND by rat isolated atria, and incubation of the atria with L-NAME, but 1H-[1,2,4]Oxadiazolo[4,3-a]quinoxalin-1-one (ODQ; inhibitor of soluble guanylyl cyclase) does not cause a fall in spontaneous atrial rate (Britto-Júnior et al., 2022a). This fall has been attributed to the inhibition of 6-ND synthesis/release, and the fall in heart rate from L-NAME chronically treated animals could be due to this mechanism. In contrast to NA and ADR, this increase in heart rate by either 6-ND or DA was abolished by chronic L-NAME treatment. Whether this is due to inhibition of NO synthesis or to hypertension development is not clear. Although we do not have a satisfactory explanation for this finding, one interesting hypothesis is that 6-ND, like DA, could be a substrate for both MAO-A and MAO-B (Shih et al., 1999). S-Nitroso-N-acetylpenicillamine (0.4–4 μM) inhibits MAO activity in rat mitochondrial homogenates (Muriel and Pérez-Rojas, 2003), indicating that NO can modulate MAO activity. Whether L-NAME treated animals present higher MAO activity is under current investigation. The finding that 6-ND, in contrast to NA and ADR, loses its positive chronotropic effect in L-NAME treated animals reinforces the concept that the transduction mechanism does not occur via adrenoceptor activation. The fact that the positive chronotropic effect of 6-ND *in vivo*, and very likely the positive inotropic effect too, is not accompanied by increases in blood pressure, supports the therapeutic potential of 6-ND for the treatment of both acute and chronic heart failure (Zatz and De Nucci, 2024).

In conclusion, the results obtained with the anaesthetized rat extend the previous observations in the rat isolated right atrium (Britto-Júnior et al., 2022a; Britto-Júnior et al., 2023c) and Langendorff preparation (Britto-Júnior et al., 2023a) that 6-ND is the most potent endogenous positive chronotropic agent yet described, and the mechanism of action is independent of MAO inhibition. The finding that the inhibition of NO synthesis by L-NAME abolished the chronotropic effect induced by 6-ND, but it did not affect that induced by ADR and NA, reinforces the concept that the mechanism of action responsible for the chronotropic effect induced by 6-ND is independent of adrenoceptor activation.

## Data Availability

The original contributions presented in the study are included in the article/Supplementary Material, further inquiries can be directed to the corresponding author.
