# Supplementary material for: Cardiovascular effects of 6-nitrodopamine, adrenaline, noradrenaline, and dopamine in normotensive and hypertensive rats
Source: Front Pharmacol. 2025 May 20;16:1557997. doi: 10.3389/fphar.2025.1557997 (PMC12129799; doi:10.3389/fphar.2025.1557997)
Supplement: Supplementary file 1 [file DataSheet1.pdf]

Supplementary Material

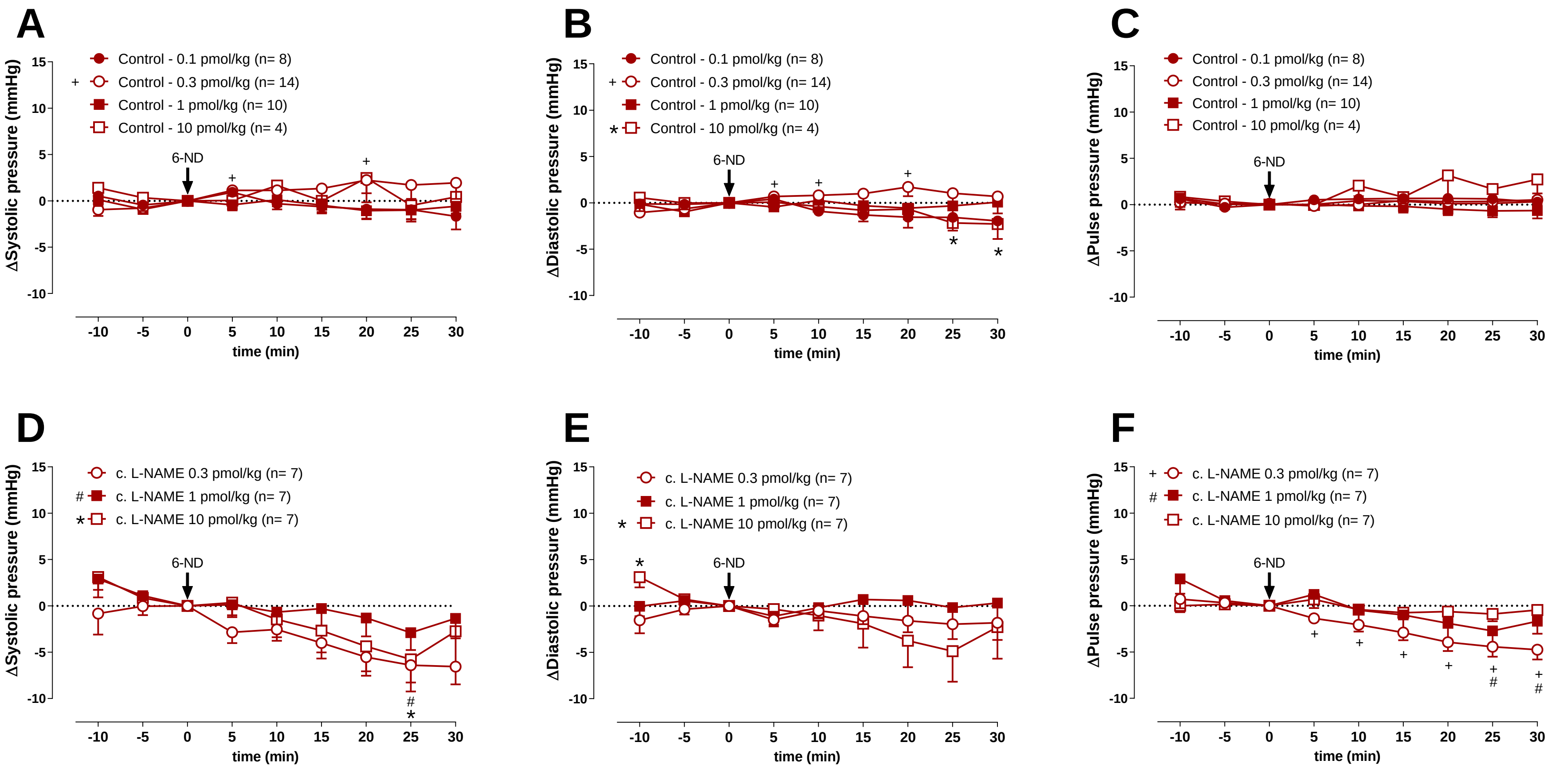

Figure S1. Changes of Systolic, Diastolic and Pulse pressure induced by intravenous bolus injections of 6-nitrodopamine (6-ND; 0.1 – 10 pmol/kg), in anesthetized control (Panel A – C) and chronically treated with L-NAME (Panel D – E) rat. The characters "+, # and *" and their position, above or below the x-axis, indicate $p < 0.05$ in comparison with the point "0", when the drug was injected. Paired Student's t-test was used to compare the basal values each five minutes after bolus administration at the same dose of 6-ND for each Panel.

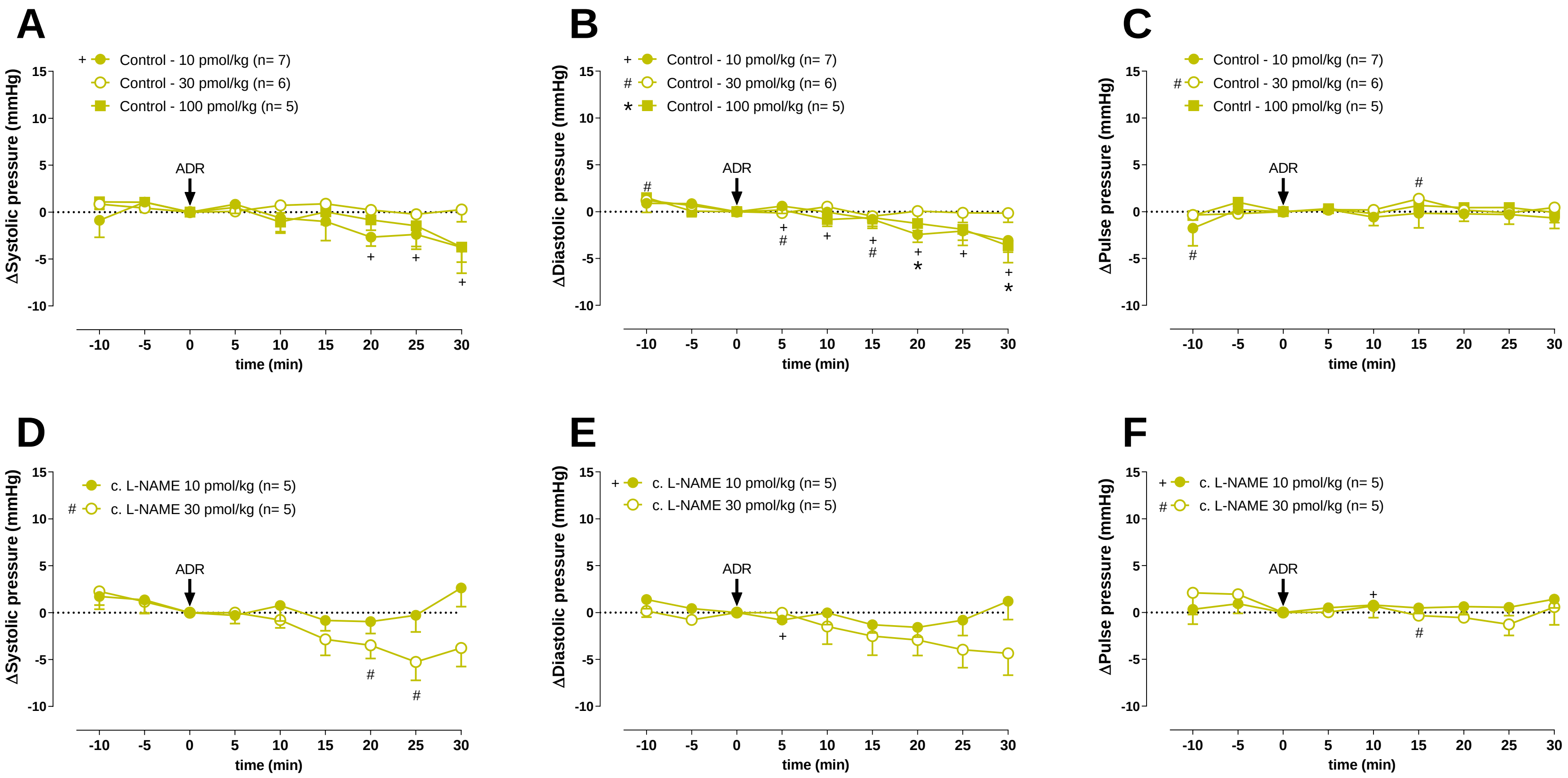

Figure S2. Changes of Systolic, Diastolic and Pulse pressure induced by intravenous bolus injections of Adrenaline (ADR; 10 – 100 pmol/kg), in anesthetized control (Panel A – C) and chronically treated with L-NAME (Panel D – E) rat. The characters "+, # and *" and their position, above or below the x-axis, indicate $p < 0.05$ in comparison with the point "0", when the drug was injected. Paired Student's t-test was used to compare the basal values each five minutes after bolus administration at the same dose of ADR for each Panel.

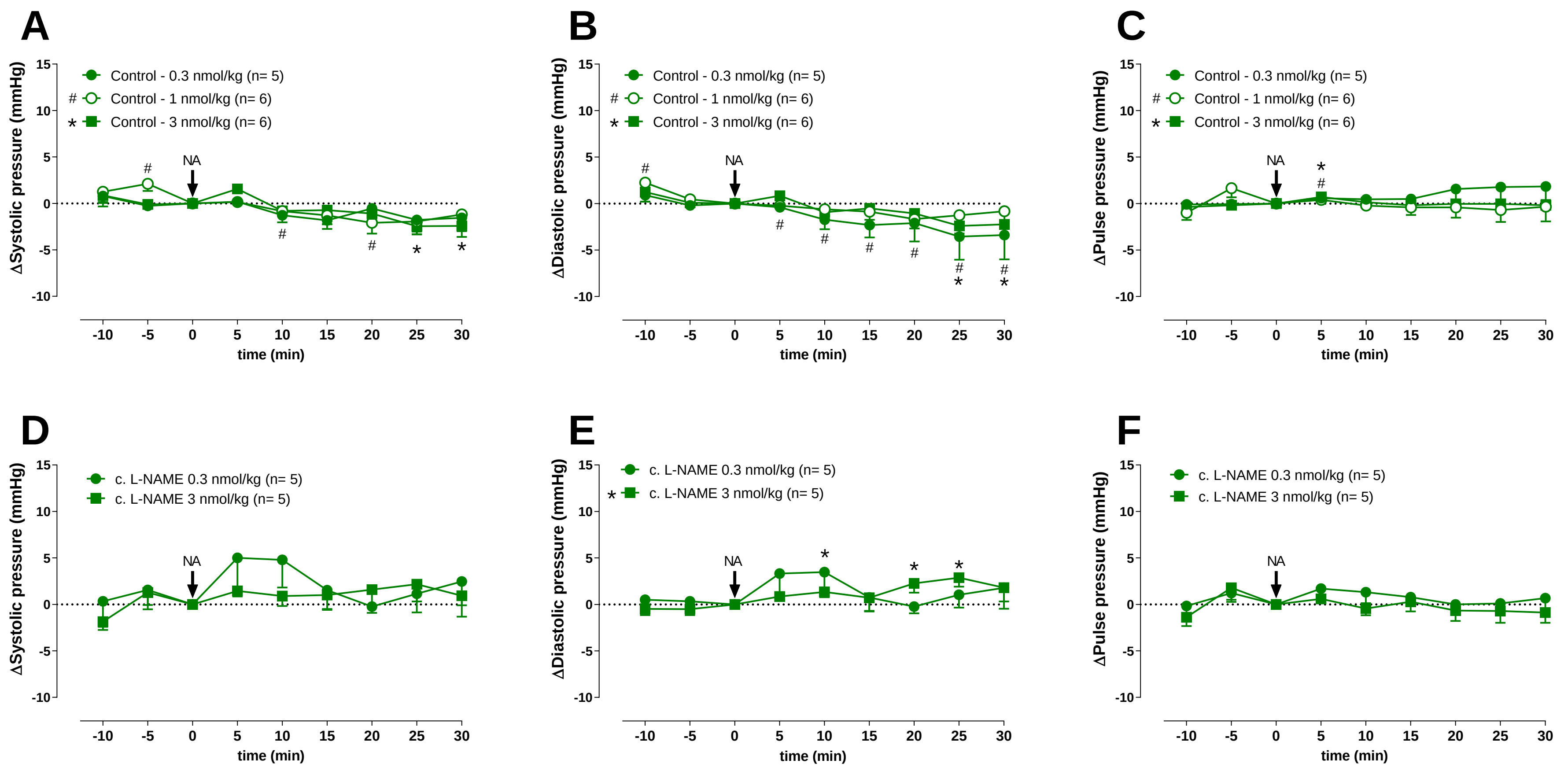

Figure S3. Changes of Systolic, Diastolic and Pulse pressure induced by intravenous bolus injections of Noradrenaline (NA; 0.3 – 3 nmol/kg), in anesthetized control (Panel A – C) and chronically treated with L-NAME (Panel D – E) rat. The characters "# and *" and their position, above or below the x-axis, indicate p< 0.05 in comparison with the point "0", when the drug was injected. Paired Student's t-test was used to compare the basal values each five minutes after bolus administration at the same dose of NA for each Panel.

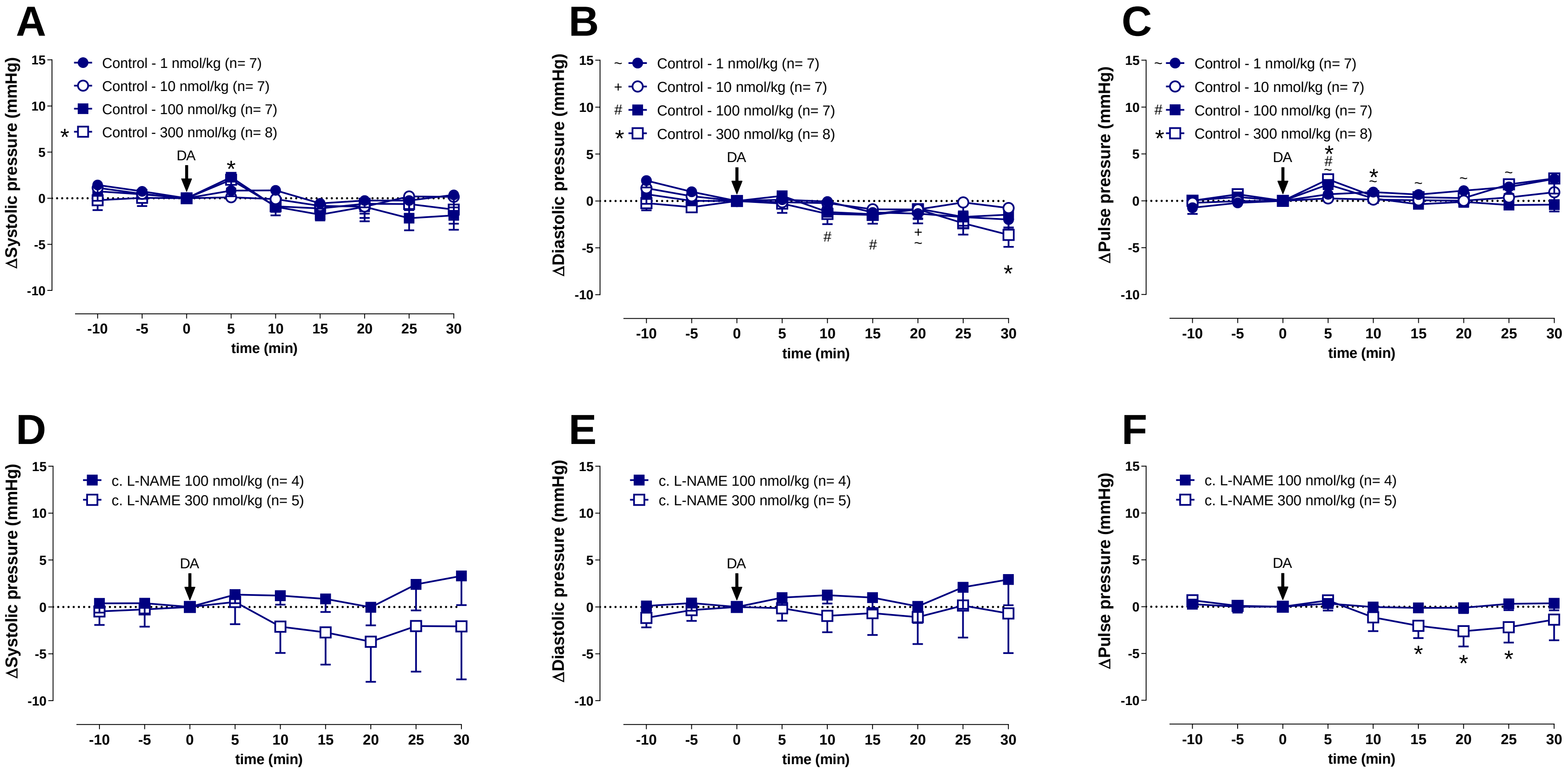

Figure S4. Changes of Systolic, Diastolic and Pulse pressure induced by intravenous bolus injections of Dopamine (DA; 1 – 300 nmol/kg), in anesthetized control (Panel A – C) and chronically treated with L-NAME (Panel D – E) rat. The characters "~, +, # and *" and their position, above or below the x-axis, indicate $p< 0.05$ in comparison with the point "0", when the drug was injected. Paired Student's t-test was used to compare the basal values each five minutes after bolus administration at the same dose of DA for each Panel.

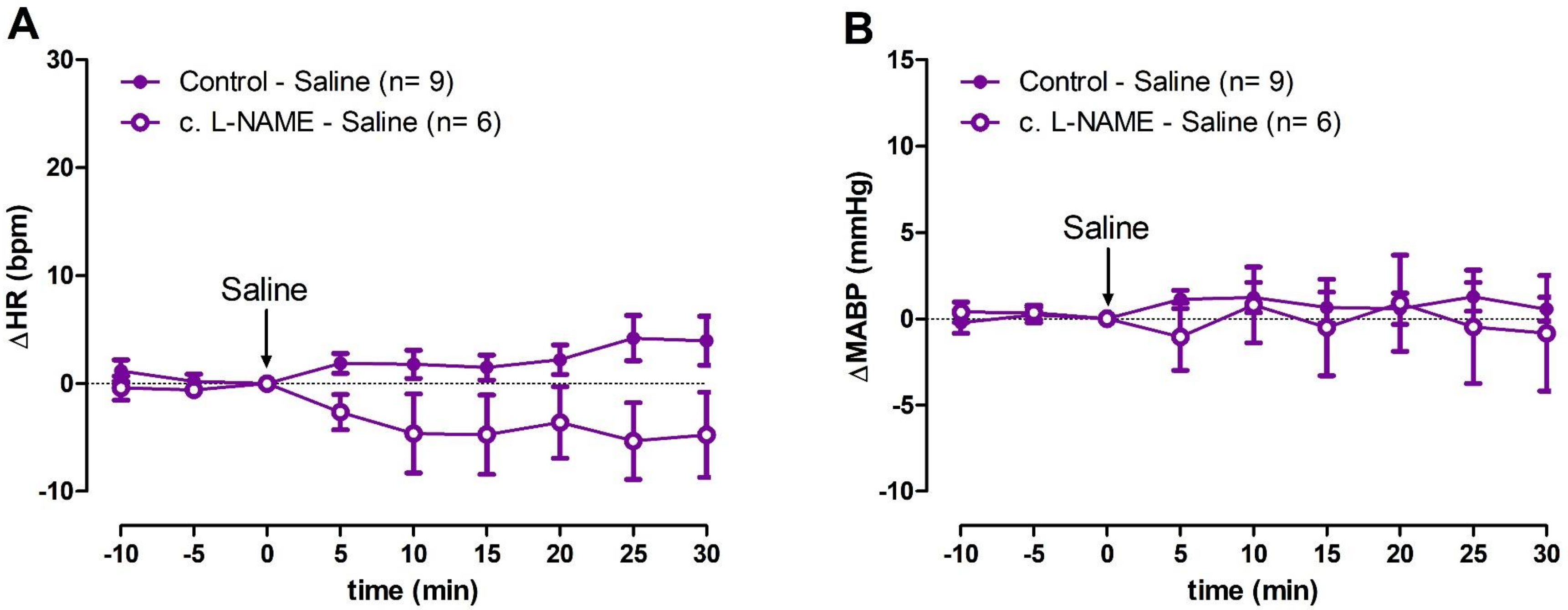

Figure S5. Changes in heart rate (Panel A, DDHR) and mean arterial blood pressure (Panel B, DMABP) by bolus injection with Saline (25µL) in anesthetized control and chronically treated with L-NAME rat. Paired Student's t-test was used to compare the basal values each five minutes after bolus administration for control and chronically treated with L-NAME rats for each Panel.

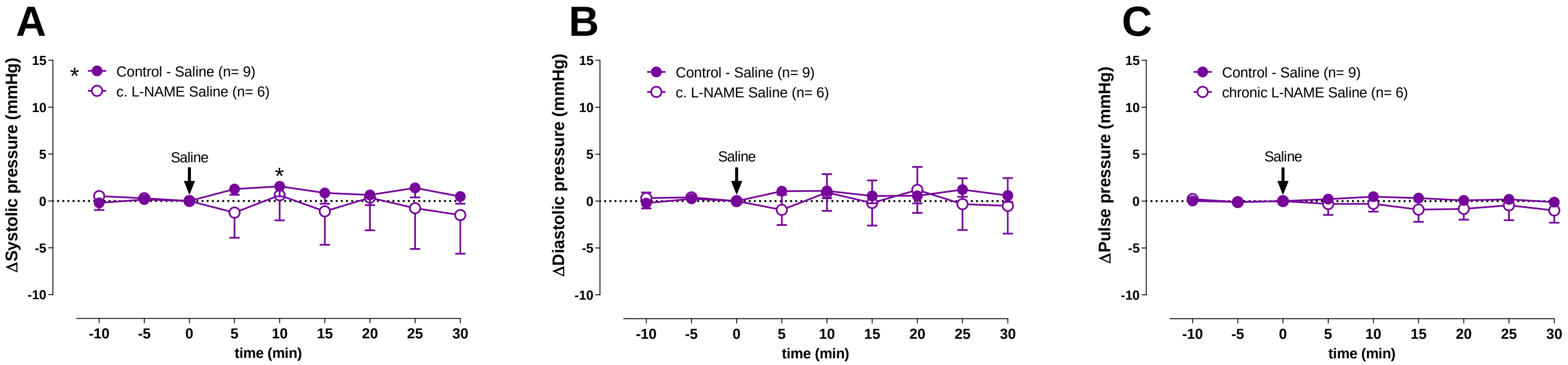

Figure S6. Changes of Systolic (Panel A), Diastolic (Panel B) and Pulse pressure (Panel C) induced by intravenous bolus injections of Saline (25µL), in anesthetized control and chronically treated with L-NAME rat. The character "*" and its position, above the x-axis, indicate $p < 0.05$ in comparison with the point "0", when the saline was injected. Paired Student's t-test was used to compare the basal values each five minutes after bolus administration for control and chronically treated with L-NAME rats for each Panel.

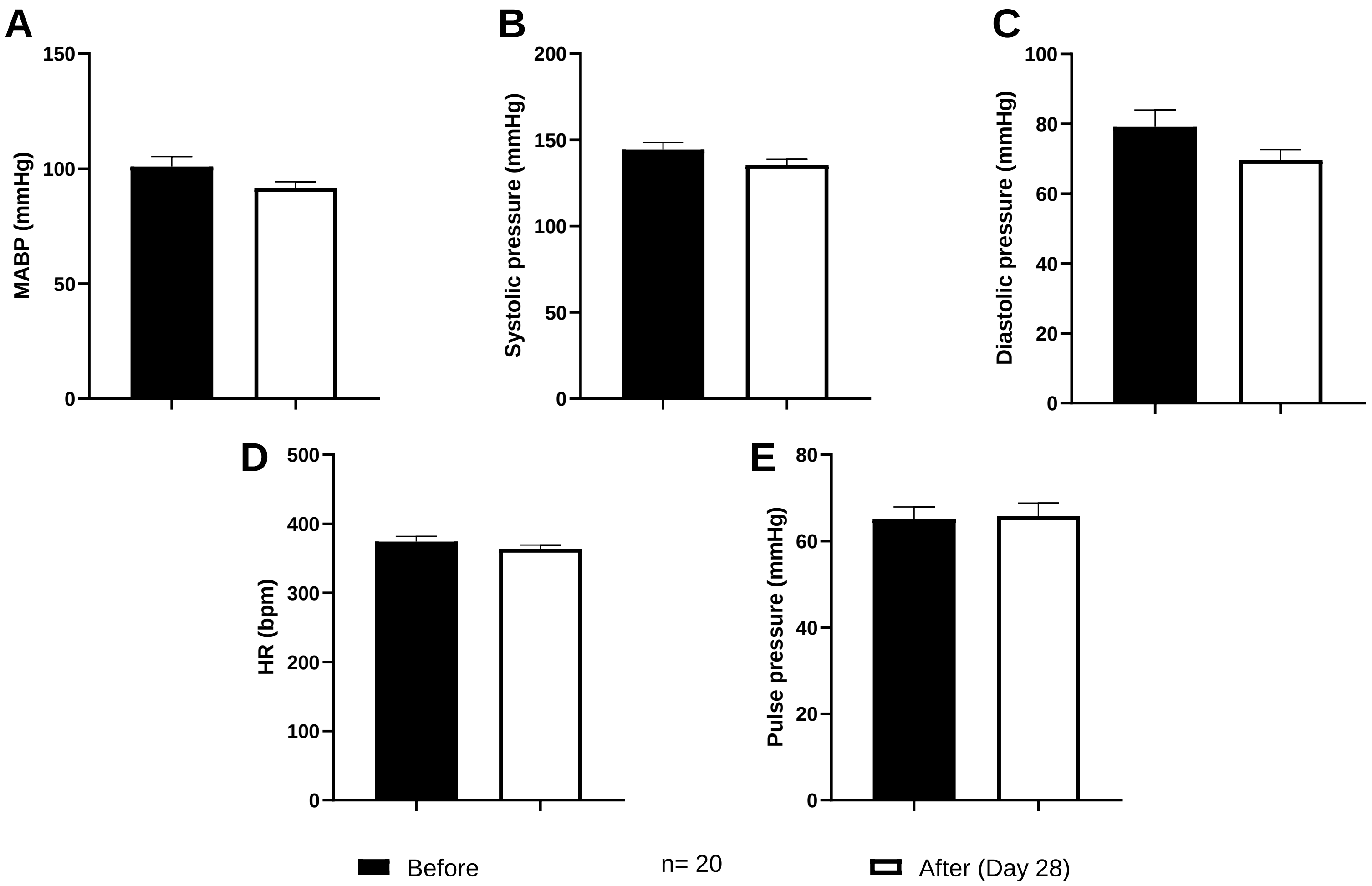

Figure S7. Measurement of the Mean Arterial Blood Pressure (Panel A; MABP), Systolic Pressure (Panel B), Diastolic Pressure (Panel C), Heart Rate (Panel D), and Pulse Pressure (Panel E) of conscious male control Wistar rats, before and after 28 days. Paired Student's t-test was used to compare the parameters before and after L-NAME chronical treatment for each Panel. Paired Student's t-test was used to compare the parameters before and after L-NAME chronical treatment for each Panel.

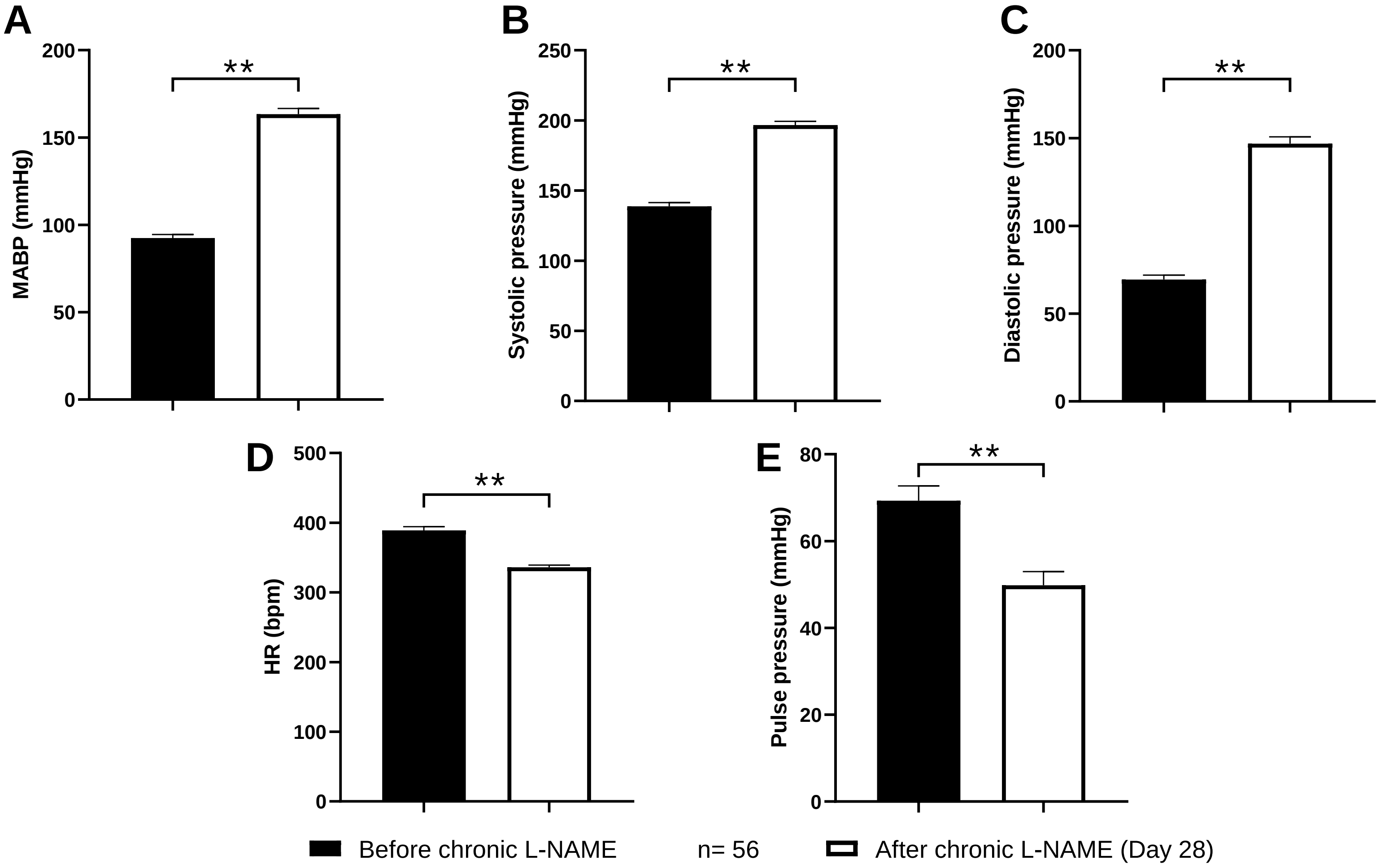

Figure S8. Measurement of the Mean Arterial Blood Pressure (Panel A; MABP), Systolic Pressure (Panel B), Diastolic Pressure (Panel C), Heart Rate (Panel D), and Pulse Pressure (Panel E) of conscious male Wistar rats, before and after treatment with chronic L-NAME (28 days). **indicates $p < 0.001$. Paired Student's t-test was used to compare the parameters before and after L-NAME chronical treatment for each Panel.
